# Supplementary figures and images for: Protein C Pretreatment Protects Endothelial Cells from SARS-CoV-2-Induced Activation
Source: Viruses. 2024 Jun 28;16(7):1049. doi: 10.3390/v16071049 (PMC11281670; doi:10.3390/v16071049)

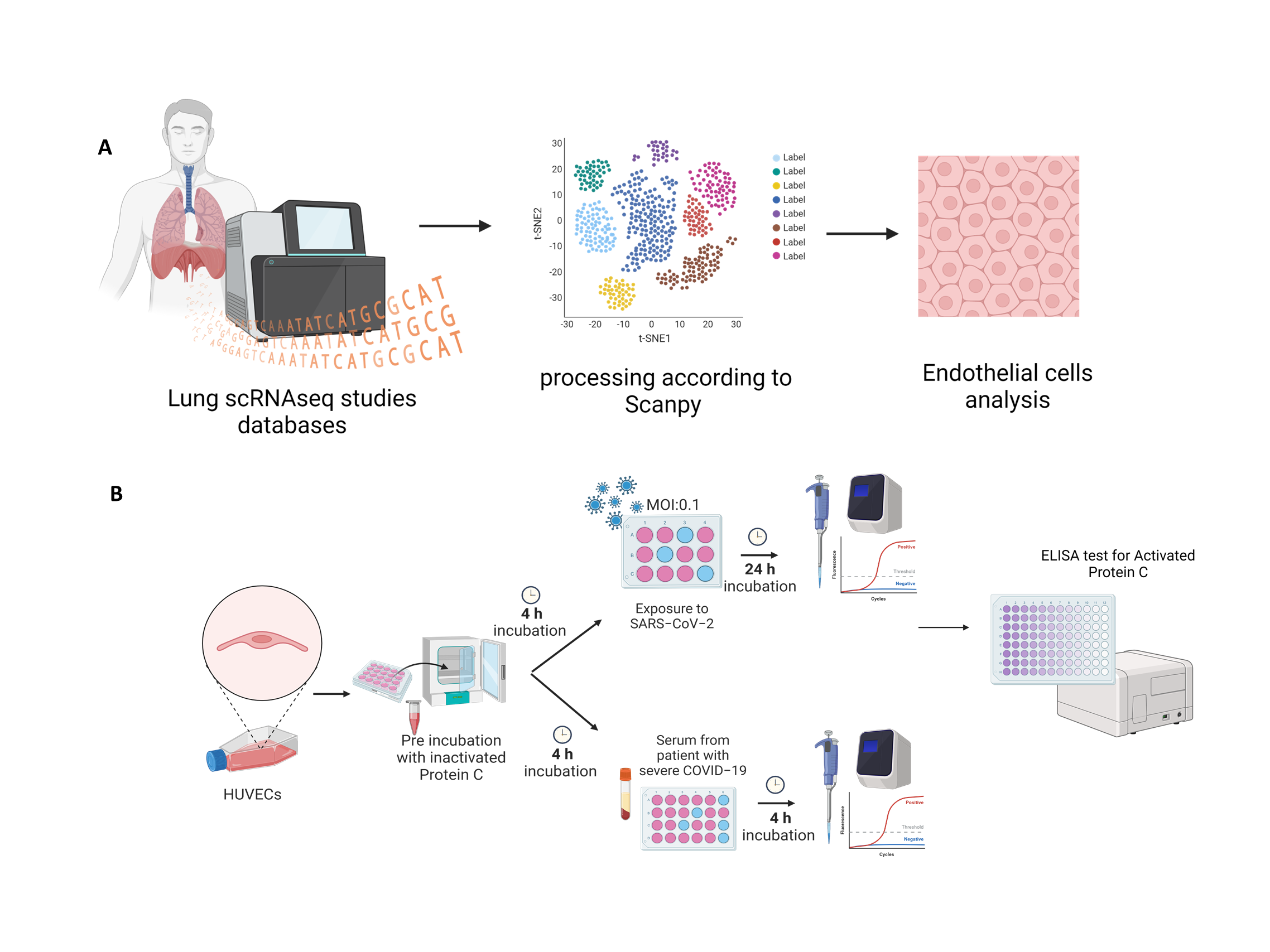

Supplement: Supplementary file 1 [file viruses-16-01049-s001.zip › figure1 Proofreading.tiff]

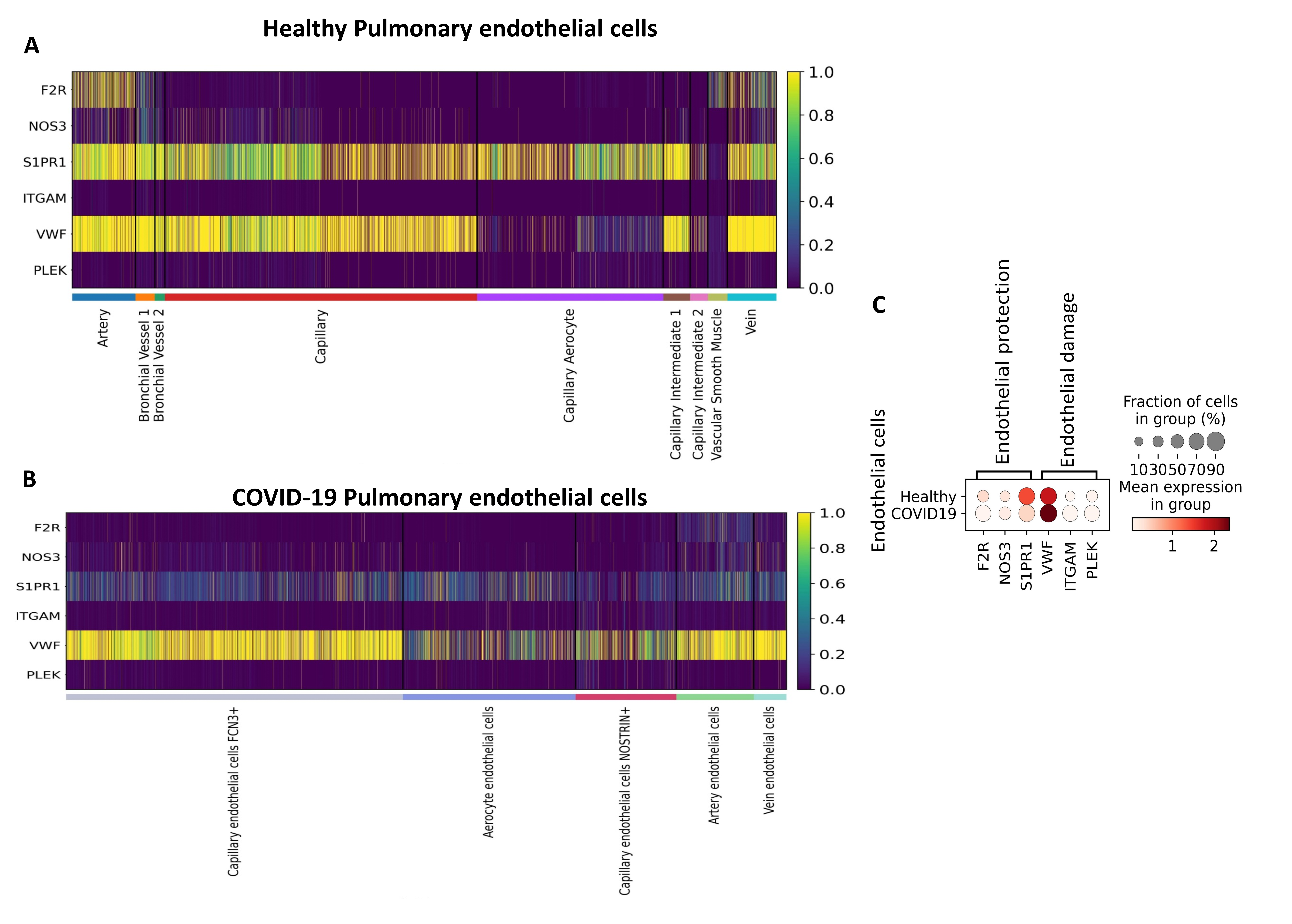

Supplement: Supplementary file 1 [file viruses-16-01049-s001.zip › figure2 Proofreading.tiff]

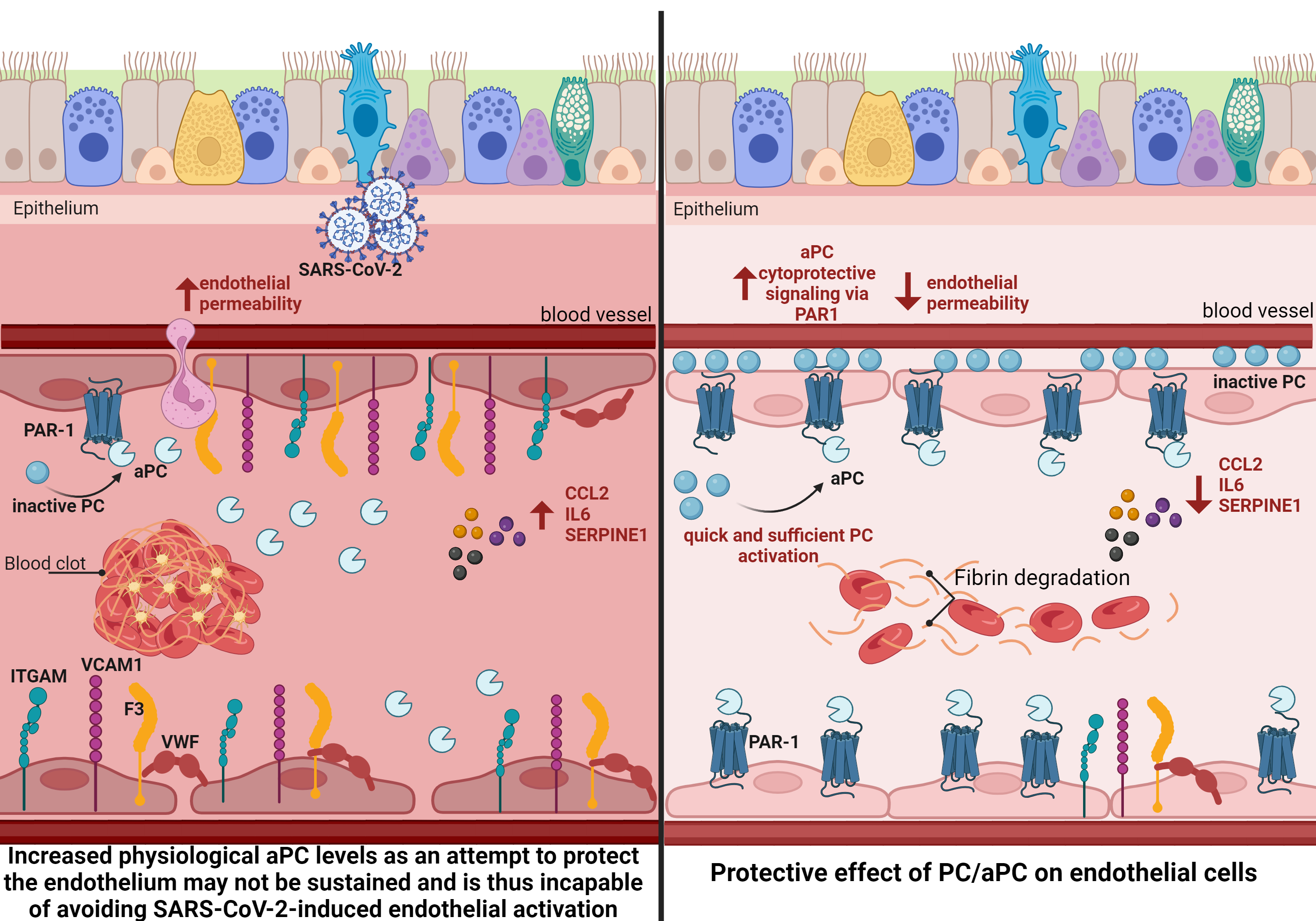

Supplement: Supplementary file 1 [file viruses-16-01049-s001.zip › figure5 Proofreading.tiff]
